# Removal of Loose Substances from the Knee Joint

**Published:** 1829-03

**Authors:** Charles Averill

**Affiliations:** Surgeon


### II. Analytical.

16. *Removal of loose substances from the knee joints.* By Charles Averill, Esq. Surgeon.

It frequently happens, that the laced bandage recommended by Mr. Hey, to confine loose masses of cartilage to one spot in the joint, are inefficient; and the extraction of them is then necessary. In the usual mode of operating, however, an excessive quantity of synovia is sometimes lost, and as the body floats from one part to another, there is no certainty of our being able to extract it through the incision which may be made for that purpose.

Mr. Averill (London Medical and Physical Journal,) has ingeniously contrived to obviate these difficulties. He found that by placing around the cartilage, when it was protruded from the joint, an iron ring, he could effectually confine it to one spot, and by cutting upon it, within the ring, could with certainty extract it, with a very inconsiderable loss of synovia. Still further, to obviate such a loss, and exclude the external air from the cavity of the joint, he advises that the skin should be pulled aside as far as possible, so that when the operation is finished, the incision into that part will not lie over that in the capsular ligament. Mr. A. has published a case in which his operation was perfectly successful.

